# Pan-American Medical Congress

**Published:** 1893-08

**Authors:** T. J. Bell

**Affiliations:** Vice-President for Texas, Tyler, Texas


### Society Notes.

### PAN-AMERICAN MEDICAL CONGRESS.

TYLER, Texas, July 15, 1893.

*To the Medical Profession of Texas:*

I will be glad to send up the application of any and all practitioners of regular medicine in good standing to join the Pan-American Medical Congress, which convenes in Washington, D. C., on the 5th day of September next, and holds four days.

You may send your name with the registration fee, $10, to myself or to Dr. A. M. Owen, Treasurer of the Congress, at Evansville, Ind., and your name will at once be enrolled as a member, and you will be entitled to all the privileges and benefits of the Congress.

There is a grand opportunity to meet face to face many of the great lights in our profession, and hear read and discussed papers on many different medical subjects by such men as Wm. Pepper, N. S. Davis, Joseph Price, S. E. Chaille, A. Loomis, and others of our own United States, as well as many distinguished men of foreign countries.

All progressive men in the medical profession should avail themselves of this opportunity at once as the time is limited.

I will gladly give any information I can in regard to the meeting, etc. T. J. BELL, M. D.,
Vice-President for Texas, Tyler, Tex.

